# Oxidative stress and DNA alteration on the earthworm *Eisenia fetida* exposed to four commercial pesticides

**DOI:** 10.1007/s11356-024-33511-7

**Published:** 2024-05-14

**Authors:** Tommaso Campani, Silvia Casini, Andrea Maccantelli, Filippo Tosoni, Antonella D’Agostino, Ilaria Caliani

**Affiliations:** 1 Department of Physical, Earth and Environmental Sciences, University of Siena, Via Mattioli, 4, 53100 Siena, Italy; 2 Department of Economics and Statistics, University of Siena, Piazza S. Francesco, 7, 53100 Siena, Italia

**Keywords:** Fungicide, Biomarkers, *E. fetida*, Genotoxic effect, Pesticide, Toxicological risk, Pesticide product formulation

## Abstract

Modern agriculture is mainly based on the use of pesticides to protect crops but their efficiency is very low, in fact, most of them reach water or soil ecosystems causing pollution and health hazards to non-target organisms. Fungicide triazoles and strobilurins based are the most widely used and require a specific effort to investigate toxicological effects on non-target species. This study evaluates the toxic effects of four commercial fungicides Prosaro® (tebuconazole and prothioconazole), Amistar®Xtra (azoxystrobin and cyproconazole), Mirador® (azoxystrobin) and Icarus® (Tebuconazole) on *Eisenia fetida* using several biomarkers: lipid peroxidation (LPO), catalase activity (CAT), glutathione S-transferase (GST), total glutathione (GSHt), DNA fragmentation (comet assay) and lysozyme activity tested for the first time in *E. fetida*. The exposure to Mirador® and AmistarXtra® caused an imbalance of ROS species, leading to the inhibition of the immune system. AmistarXtra® and Prosaro®, composed of two active ingredients, induced significant DNA alteration, indicating genotoxic effects. This study broadened our knowledge of the effects of pesticide product formulations on earthworms and showed the need for improvement in the evaluation of toxicological risk deriving from the changing of physicochemical and toxicological properties that occur when a commercial formulation contains more than one active ingredient and several unknown co-formulants.

## Introduction

Modern agriculture uses thousands of tons of pesticides to protect crops and to increase production and food storage (Chen et al. 2018). The efficiency of pesticides is very low, only 0.1% of them reach the target organism, whereas the rest of them reach water or soil ecosystems, causing possible severe environmental pollution and health hazards to non-target organisms and ecosystems (Ma et al. 2019). For this reason, pesticides have a central responsibility for terrestrial biodiversity declines (Brühl and Zaller 2019). One of the classes of pesticides most used in agriculture is fungicides which defend crops from attack by moulds that produce mycotoxins that represent a risk to animal and human health aiming to improve crop quality and health. Among the different fungicides’ active ingredients commercialised, triazoles and strobilurins are the most widely used worldwide, requiring then a specific effort to investigate potential toxicological effects on non-target species (Bartlett et al. 2002; Wang et al. 2021). The active ingredient azoxystrobin belongs to the strobilurin family and is the main active ingredient in the product formulation of Amistar^®^Xtra (200 g/L) in co-formulation with cyproconazole (80 g/L), while it is the only active ingredient of Mirador^®^ (250 g/L). Its half-life values are 107.47 and 62.69 days in aerobic and anaerobic soil respectively (Ghosh and Singh 2009). Azoxystrobin and cyproconazole are “extremely toxic” to *E. fetida* earthworms (48 h LC_50_ = 2.72 and 8.48 μg/cm^2^, respectively) in filter paper contact test and “acutely toxic” in OECD soil test (14d LC_50_ 327.4, 211.8 mg/kg (Wang et al. 2012). Tebuconazole alone and tebuconazole and propiconazole are the main active ingredients in the product formulation of Icarus^®^ (200 g/L) and Prosaro^®^ (125 g/L), respectively. These active ingredients belong to the triazoles family, and both are widely used against various foliar diseases in cereals and other field crops. Due to its high persistence in soil (49–610 days), tebuconazole negatively affects the soil’s biological properties (Chen et al. 2018; Baćmaga et al. 2019). Tebuconazole is classified as very toxic for *Eisenia fetida* earthworm (48 h LC_50_ = from 5.7 to 31.57 μg/cm^2^; Chen et al. 2018), based on a filter paper contact test and acutely toxic in the OECD soil test (14d LC_50_ 895.2 mg/kg; Wang et al. 2012). The commercial formulations of these pesticide product formulations (PPFs) are composed of one or more active ingredients and other substances labelled as “inerts”, “adjuvants” or “co-formulants”. These substances are added usually to make the PPF polar and more stable, to improve the absorption by the target organisms (Nagy et al. 2020). The co-formulants, which are supposedly “inert”, have biological activity on their own and may be toxic to non-target organisms even more than the active ingredients (Wagner et al. 2015; Adams et al. 2021). Furthermore, the adjuvants could have synergistic effects with the active ingredients and amplify their toxicological effects (Cox and Surgan 2006). Producer companies do not declare the exact composition of pesticide co-formulants because often specific molecules, or the formula itself, are covered by a patent. Moreover, this lack of information about the composition of PPFs could be potentially dangerous to human health. In European legislation, the risks of PPFs are evaluated only based on the toxicity of the active ingredients (EU 1107/2009), and co-formulants individually, but ignore the possible toxicological effects of the mix of active ingredients and co-formulants present in the commercial formulation (Nagy et al. 2020). To our knowledge, several papers investigated the toxicological effects of tebuconazole, propiconazole, cyproconazole and azoxystrobin on non-target organisms such as earthworms (Han et al. 2014; Hackenberger et al. 2018; Xu et al. 2021; Wu et al. 2021), fish (Han et al. 2016), daphnia and algae (Zhang et al. 2020) or cultured cells (Schwarzbacherová et al. 2015) but only a few authors investigated the effect of the PPFs (Leitão et al. 2014; Rico et al. 2016; Bart et al. 2017; Gomes et al. 2021; Jorge-Escudero et al. 2022). The lack of information about the chemical composition of commercial fungicides and the scarce data about the toxicity of this type of formulation represents a gap to be filled so that awareness regarding the use of pesticides is raised in agriculture. The evaluation of sub-lethal endpoints, which are precursors of long-term individual and population-level effects can be carried out by the use of biomarkers. Biomarkers are sensitive and useful tools based on biochemical, cellular and behavioural tests developed to investigate the sub-lethal effects of contaminants on organisms. This approach is complementary to standard toxicity tests and provides more information about the organism’s stress responses and the toxic mode of action of the pesticide (Gastaldi et al. 2007; Rico et al. 2016) leading to a better understanding of anthropogenic stressors such as PPFs (Campani et al. 2017; Caliani et al. 2021). Biomarkers are also an early warning system able to prevent toxicological effects at higher biological level. Earthworms are simple model organisms considered useful bioindicators of chemical toxicity in the terrestrial ecosystem (Pelosi et al. 2014; Liu et al. 2017). In particular, *E. fetida* (Savigny, 1826) is a suitable model species for ecotoxicological testing because of the standardisation of its acute and chronic ecotoxicity assays (Amossé et al. 2020) and the ease of breeding and maintenance. This study aimed to evaluate the potentially toxic effects of four commercial fungicides (Prosaro®, Amistar®Xtra, Mirador® and Icarus®) on the earthworm *E. fetida* using a biomarker approach. Specimens of *E. fetida* were exposed for 24 h to different fungicides by filter paper test experiments. On the *E. fetida* specimens, we evaluated a battery of enzymatic and cellular biomarkers to investigate the presence of oxidative stress by lipid peroxidation (LPO), catalase activity (CAT), glutathione S-transferase (GST) and total glutathione (GSHt). Alteration of the immune system was tested for the first time by the lysozyme activity (LYS) and genotoxic effects were tested by the quantification of DNA fragmentation (comet assay).

## Materials and methods

### Bioindicators

*Eisenia fetida* (Savigny 1826) adults, obtained from a commercial earthworm breeding farm (Lombricoltura Compagnoni, Mandello sul Lario, Como, Italy), were maintained in the laboratory culture at 25 °C for 4 weeks before the start of the experiments and fed with organic manure. *E. fetida* well-clitelled adults were placed on moist paper until they expelled all the gut content (1h maximum) after which they were washed with distilled water, dried and prepared for the filter paper test.

### Fungicide

Four commercial fungicides, widely used in cereal crops defence, were selected: Prosaro®, Bayer crop science Italy (tebuconazole 125 g/L and prothioconazole 125 g/L), Amistar®Xtra, Syngenta, Italy (azoxystrobin 200 g/L and cyproconazole 80 g/L), Mirador®, Adama Italy (azoxystrobin 250 g/L) and Icarus®, Adama Italy (tebuconazole 200 g/L). The stock solutions were prepared by diluting the commercial products in distilled water.

Two doses for each fungicide (Table 1) were calculated and prepared following the recommended field rate for wheat crops as reported on the product labels. The high dose corresponds to the field application rate.

**Table 1:** Treatments dose of the commercial fungicides tested. The high dose corresponds to the field application rate

Treatment	Amistar® Xtra	Mirador®	Prosaro®	Icarus®
Control	0 μg/cm^2^	0 μg/cm^2^	0 μg/cm^2^	0 μg/cm^2^
Low dose	1 μg/cm^2^	2 μg/cm^2^	0.75 μg/cm^2^	1 μg/cm^2^
High dose	2 μg/cm^2^	2.5 μg/cm^2^	1.25 μg/cm^2^	2 μg/cm^2^

### Sub-lethal concentrations exposure

The toxicity tests were performed according to the OECD guideline by the filter paper test (FPT). A total of 24 *E. fetida* specimens for each commercial fungicide (8 per treatment group) were exposed. Each animal was exposed in a 9cm Petri dish lined with a piece of filter paper without overlapping. The full battery of biomarkers was tested on each animal.

### Sampling

After 24 h of exposure, the viability of the animals was assessed based on the operator’s observation, using a scale from zero to three based on the individual’s mobility (0 for the deceased individual, 1 for barely perceptible movements, 2 for clear movements of the body, 3 for no movement deficit).

The coelomocytes were obtained by extrusion following the method of Eyambe et al. (1991). After the extrusion, the cell suspension was first centrifuged at 150 × *g* at 4 °C for 2 min to remove mucus, and then for 10 min to recover cells for the comet assay. Animals were sacrificed by immersion in cold nitrogen.

### Sample preparation

Animals were homogenised with a Potter homogeniser in 0.1 M K phosphate buffer (1:10 w:v). An aliquot of homogenised tissue was used for lipid peroxidation. The remaining homogenate was centrifuged at 13,200 × *g* for 20 min at 4 °C and the post-mitochondrial fraction (PMS) supernatant was removed and used for the determination of glutathione S-transferase, catalase and lysozyme activity. To the determination of the total glutathione, the protein content of the PMS fraction was precipitated with trichloroacetic acid (12%) for 1 h and then centrifuged at 13,400 × *g* for 5 min (4 °C).

### Oxidative stress

#### Lipid peroxidation (LPO)

Lipid peroxidation (LPO) was estimated using the procedure of Ohkawa et al. (1979) and Bird and Draper (1984), with some modifications. Five microliters of 4% butylated hydroxytoluene (BHT), was mixed with 150 μL of homogenate. One hundred microliters of the homogenate and BHT was mixed with 1 mL of 12% trichloroacetic acid (TCA), 0.90 mL Tris–HCl (60 mM, pH 7.4 and 0.1 mM DPTA) and 1 mL of 0.73% 2-thiobarbituric acid (TBA). The mixture was heated for 1 h at 100 °C and then cooled down to room temperature and centrifuged at 13,400 × *g* for 5 min. Each sample was analysed in duplicate. Absorbance was measured at 535 nm with AGILENT Cary UV 60 spectrophotometer and LPO expressed as nmol of thiobarbituric acid reactive substances (TBARS) formed × mg protein^−1^ (ε = 1.56 × 10^5^ M cm^−1^).

#### Glutathione S-transferase (GST)

Glutathione S-transferase activity was measured in PMS fraction according to Habig et al. (1974). Briefly, the enzyme activity was quantified by measuring the conjugation of reduced glutathione (GSH) with 2,4-dinitrochlorobenzene (DNCB) in a mixture with K phosphate buffer 0.2 M (pH 7.9) 0.2 mM DNCB, 0.02 mM GSH and PMS. Each sample was analysed in duplicate. Absorbance was measured at 342 nm (25 °C) and expressed as nmol DNCB × min^−1^ × mg protein^−1^ (ε = 9.6 × 10^−3^ M cm^−1^).

#### Catalase activity (CAT)

Catalase activity was measured in PMS fraction according to Aebi (1984) method by measuring the rate of hydrogen peroxide (H_2_O_2_) reduction as absorbance (240 nm) decreased over 1 min. Each sample was analysed in duplicate. CAT activity was expressed as μmol H_2_O_2_ × min^−1^ × mg protein^−1^ using an AGILENT Cary UV 60 spectrophotometer.

#### Total glutathione (GSHt)

Total glutathione (GSHt) was determined by spectrophotometry at 412 nm using glutathione reductase (GR) excess following the reaction between GSH with DTNB that produces 5-thio-2-nitrobenzoic acid (TNB) (Jollow et al. 1974). Each sample was analysed in duplicate. TNB formation was measured and the results were expressed as nmol TNB formed × min^−1^ × mg^−1^ protein (ε = 14.1 × 10^−3^ M^−1^ cm^−1^).

#### Protein concentration

Protein concentrations of each sample were measured in duplicate spectrophotometrically using the Biorad protein assay following the Bradford method (Bradford 1976).

### Immune system

#### Lysozyme activity (LYS)

The lysis of *Micrococcus lysodeikticus* was measured using the standard turbidity assay described by Keller et al. (2006) with slight modification. For each sample, 25 μL PMS was added in quadruplicate to the plate, 175 μL/well of *M. lysodeikticus* in 0.1 M phosphate buffer (pH 5.9) was quickly added to three sample wells and each of the standard wells. The fourth well containing PMS received a 175 μL phosphate buffer and served as a blank. Plates were assessed for absorbance at 450 nm with a spectrophotometer (microplate reader Model 550, BioRad) immediately (T_0_) and again after 5 min (T_5_). The result was expressed in HEL concentration (μg/μL) via linear regression of the standard curve.

### Genotoxic effects

#### Comet assay

The alkaline comet assay was performed as described by Singh et al.(1988) with slight modifications as reported in Campani et al. (2017). Cell suspension of coelomocytes of each animal was mixed with low melting-point agarose (LMA) (0.5%) and then spread onto a degreased slide previously dipped in normal melting-point agarose (NMA) (1%). The slides were immersed in the lysis solution (2.5 M NaCl, 100 mM EDTA, 10 mM Tris, 1% Triton X-100, pH 10,) for 1 h at 4 °C and then immersed for 20 min in the buffer (300 mM NaOH, 1 mM EDTA, pH > 13). Afterwards, electrophoresis was performed at 300 mA (25 V) for 10 min. The slides were neutralised with 0.4 M Tris, pH 7.5 and fixed in methanol. Fifty cells per sample were examined under the epifluorescence microscope (Olympus BX41) at 400 × magnification and quantificate as % of DNA content in the tail, by the use of the Komet 5.0 Software (Kinetic Imaging Ltd.).

### Statistical analysis

The different biomarker responses were related to the control by distribution-free procedures (non-parametric tests). To validate the use of a non-parametric approach, the Shapiro–Wilk test was conducted to check the normality of the data. The evaluation of the statistically significant differences between groups of treatment was conducted by the use of the Kruskal–Wallis equality of populations rank test. Then, Dunn’s non-parametric pairwise multiple comparisons test in independent groups was used. Finally, the Spearman rank correlation test was used to evaluate the degree of correlation between variables.

## Results and discussion

### Viability

Table 2 shows the results of the viability of *E. fetida* exposed to the four commercial fungicides by the filter paper test. The viability decreased from the control to the high dose in all tested fungicides except Icarus. The high doses of Amistar®Xtra and Mirador® and both doses of Prosaro® showed statistically significant differences to the respective control (*p* < 0.05). The effect was more marked in the Amistar®Xtra and Mirador® treatments, where the viability had a reduction of 54% and 33% respectively at the higher dose.

**Table 2:** The viability of *E. fetida.* Different letters indicate statistical differences between groups (*p* < 0.05)

Treatment	Amistar® Xtra	Mirador®	Prosaro®	Icarus®
Control	3.00 (a)	3.00 (a)	2.92 (a)	2.87 (a)
Low dose	2.25 (a)	3.00 (a)	2.12 (b)	2.50 (a)
High dose	1.37 (b)	2.00 (b)	2.12 (c)	2.75 (a)

### Mirador®

**Fig. 1:**
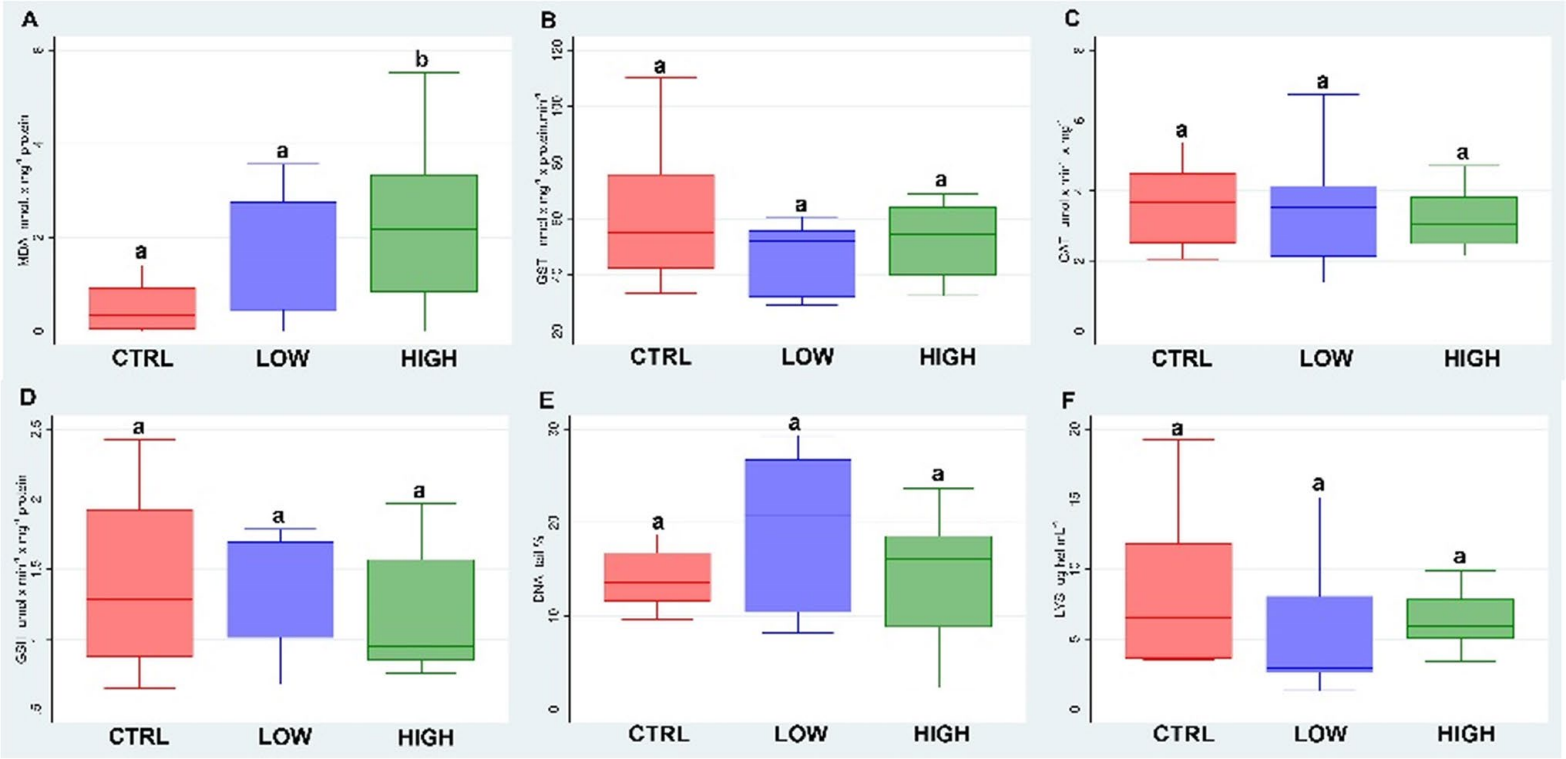
Boxplots (median, inter-quartile range and range of scores) of the biomarkers (lipid peroxidation levels (LPO) (**A**); glutathione S-transferase activity (GST) (**B**); catalase activity (CAT) (**C**); total glutathione levels (GSHt) (**D**); comet assay (**E**); lysozyme activity (LYS) (**F**) measured in *E. fetida* exposed to the fungicide Mirador®. Different letters indicate the statistical differences between groups (*p* < 0.05)

Figure 1 summarises the biomarker results of the fungicide Mirador® tested by the filter paper test. The results showed an increase in LPO (A) in both treatments with a statistical difference between the 2.5 μg/cm^2^ and the control (*p* < 0.05). No differences were observed in the GST (B), CAT (C) and GSHt (D) activity with respect to the control. Furthermore, DNA fragmentation (E) and lysozyme activity did not show any alteration in the treatment with respect to the control.

Azoxystrobin which is the active ingredient of Mirador®, has a mechanism of toxicity that inhibits respiratory fungal mitochondria from transferring electrons between cytochrome b and C_1_ and also inhibits adenosine triphosphate (ATP) synthesis. This biochemical mechanism was found to cause oxidative stress in non-target organisms such as the Diatom *Phaeodactylum tricornutum* (Du et al. 2019; Jiang et al. 2019). Antioxidant enzymes of the animal keep a balance of reactive oxygen species (ROS) and reduce oxidative damage. Han et al. (2014) report that an excess of ROS, in particular, H_2_O_2_, blocks the activity of the catalase enzyme and causes an increase in lipid peroxidation. Our LPO levels results are in line with these results and reflect the mechanism by which *E. fetida* is not able to protect itself from the toxicity of the fungicide.

Many authors report the induction of GST, an enzyme involved in the detoxification of electrophile molecules and cellular antioxidant defence mechanisms (Han et al. 2014), in different species exposed to the azoxystrobin active ingredient (Lushchak et al. 2018; Garanzini et al. 2019; Uçkun and Öz, 2021). Contrarily, our results of GST activity showed no induction in specimens exposed to Mirador®. This finding is probably due to the presence of co-formulants that modify the physicochemical characteristic of azoxystrobin that may not permit the action of GST antioxidant enzyme but induce oxidative stress.

The present study reports, for the first time, the results of lysozyme activity in *E. fetida* as biomarker for the immune system.

### Amistar®Xtra

**Fig. 2:**
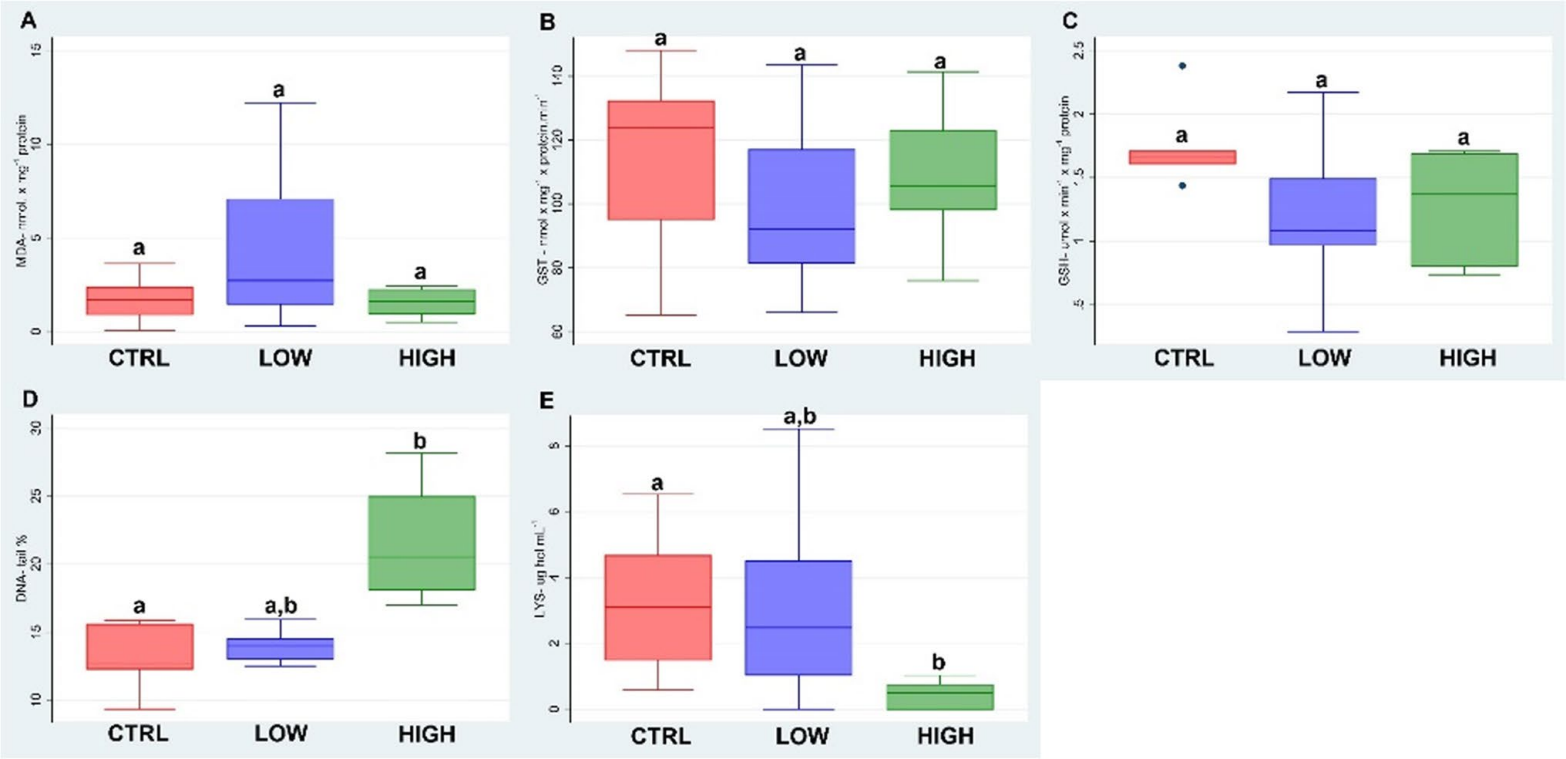
Boxplots (the median, the inter-quartile range and the range of scores) of the biomarkers (lipid peroxidation levels (LPO) (**A**); glutathione S-transferase activity (GST) (**B**); total glutathione levels (GSHt) (**C**); comet assay (**D**); lysozyme activity (LYS) (**E)**) measured in *E. fetida* exposed to the fungicide Amistar®Xtra. Different letters indicate the statistical differences between groups (*p* < 0.05)

Figure 2 summarises the biomarker results of the fungicide Amistar®Xtra tested by the filter paper test. Although there is not a statistically significant difference, the LPO (A) showed a bell-shaped trend with a decrease in the level of MDA in the treatment at the higher dose. The GST activity (B) and the GSHt levels (C) showed no differences in both doses with respect to the control. The comet assay (D) showed a statistically significant increase in DNA fragmentation at 2 μg/cm^2^ (*p* < 0.05). LYS (E) showed an inhibition in the 2 μg/cm^2^ treatment with a statistical difference with respect to the control and 1 μg/cm^2^ treatment.

Amistar®Xtra is a systemic fungicide with a double active ingredient, azoxystrobin and cyproconazole. The marked intensity of DNA damage in the highest treatment dose is probably due to the presence of an accumulation of ROS not suitably blocked by the antioxidant enzymes, as reported by various authors (Han et al. 2014; Zhang et al. 2018; Ma et al. 2019). *Eisenia fetida* exposed to Amistar®Xtra showed oxidative stress as reported by Xu et al. (2021) and Han et al. (2014) and previously described for the Mirador® treatments. This evidence was confirmed in our work by the high levels of MDA, with its bell-shaped trend with the highest concentration showing values similar to the control. This hypothesis considers the fact that the LPO values are higher in the treatments with Amistar®Xtra than in the other fungicides tested in this work and the synergistic effects of azoxystrobin and cyproconazole overwhelmed the capacity of antioxidant activity to contrast the proliferation of ROS. Furthermore, the presence of ROS reduced the functionality of lysozyme at the higher dose.

### Icarus®

**Fig. 3:**
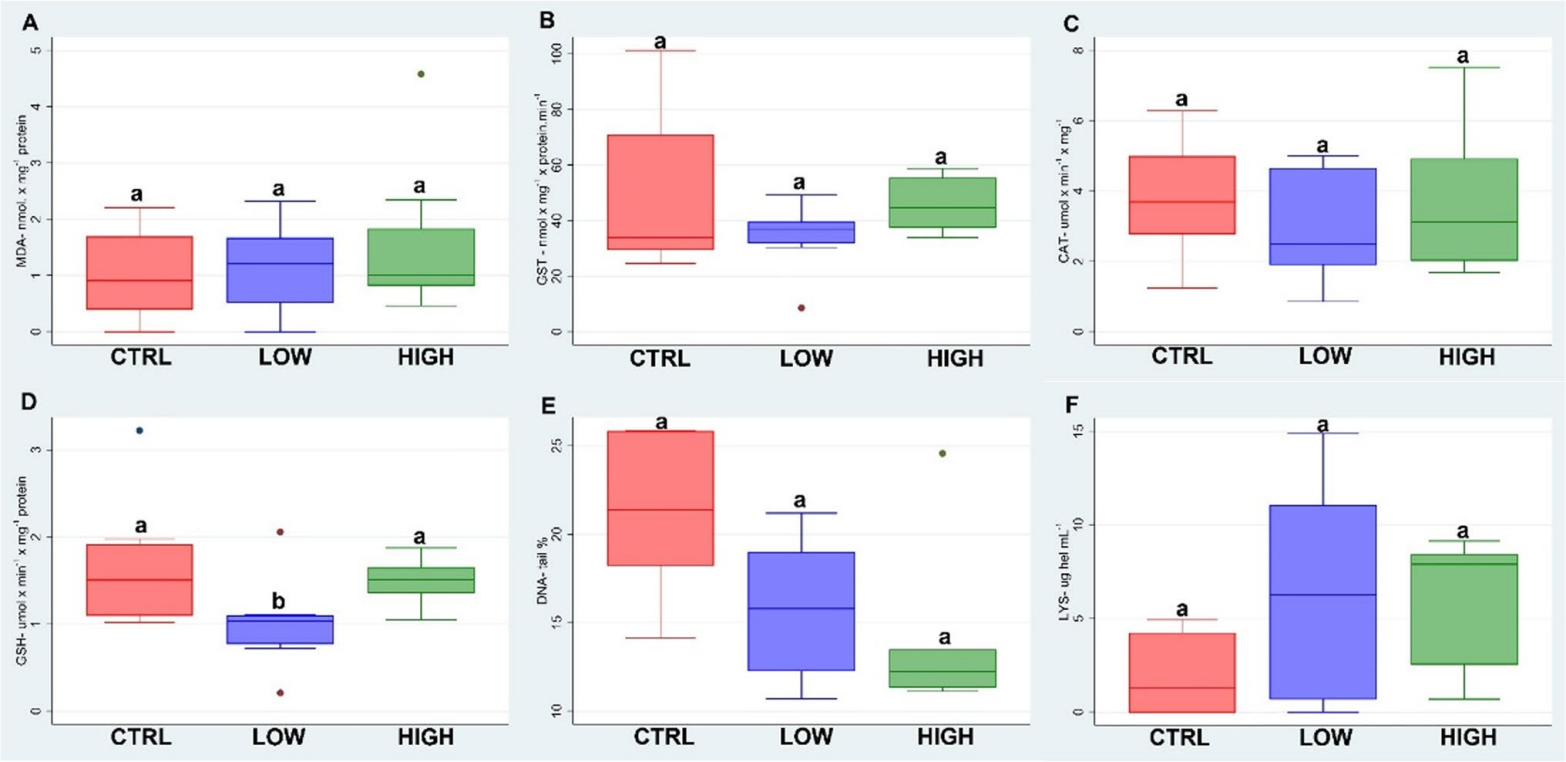
Boxplots (the median, the inter-quartile range and the range of scores) of the biomarkers (lipid peroxidation levels (LPO) (**A**); glutathione S-transferase activity (GST) (**B**); catalase activity (CAT) (**C**); total glutathione levels (GSHt) (**D**); comet assay (**E**); lysozyme activity (LYS) (**F**)) measured in *E. fetida* exposed to the fungicide Icarus®. Different letters indicate a statistical difference between groups (*p* < 0.05)

Figure 3 summarises the biomarker results of the fungicide Icarus® tested by the filter paper test. After the treatments, no significant changes in LPO levels (A), GST (B) and CAT (C) activities were observed, indicating that Icarus® did not induce oxidative stress and peroxidation of lipid membranes in the earthworms. A reduction in the GSH levels (D) was found at the treatment of 1 μg/cm^2^ with a statistical difference with respect to the control and the 2 μg/cm^2^ treatment (*p* < 0.05). The comet assay (E) showed no statistical differences between the control and treatments. LYS (F) showed high variability in the treatment without differences between both the doses and the control.

Icarus® is a wide-spectrum fungicide with the active ingredient tebuconazole. The highest concentration considered in this paper, which coincided with the field dose, was very low with respect to the LC50 of the active ingredient tebuconazole (LC_50_ of 9.4 μg/cm^2^) and did not seem to change the enzyme activity.

Icarus® seemed not to have toxicological effects at molecular and behavioural levels on *E. fetida* at field dose concentrations. A recent study by Martins et al. (2023) showed no toxicological effects in specimens of *Osmia bicornis* exposed to a tebuconazole-based commercial fungicide. However, some authors report that tebuconazole can induce tumours and developmental anomalies in mice, ocular lesions in dogs and developmental anomalies in rats and rabbits in long-term exposure (Schwarzbacherová et al. 2015; Coremen et al. 2022).

### Prosaro®

**Fig. 4:**
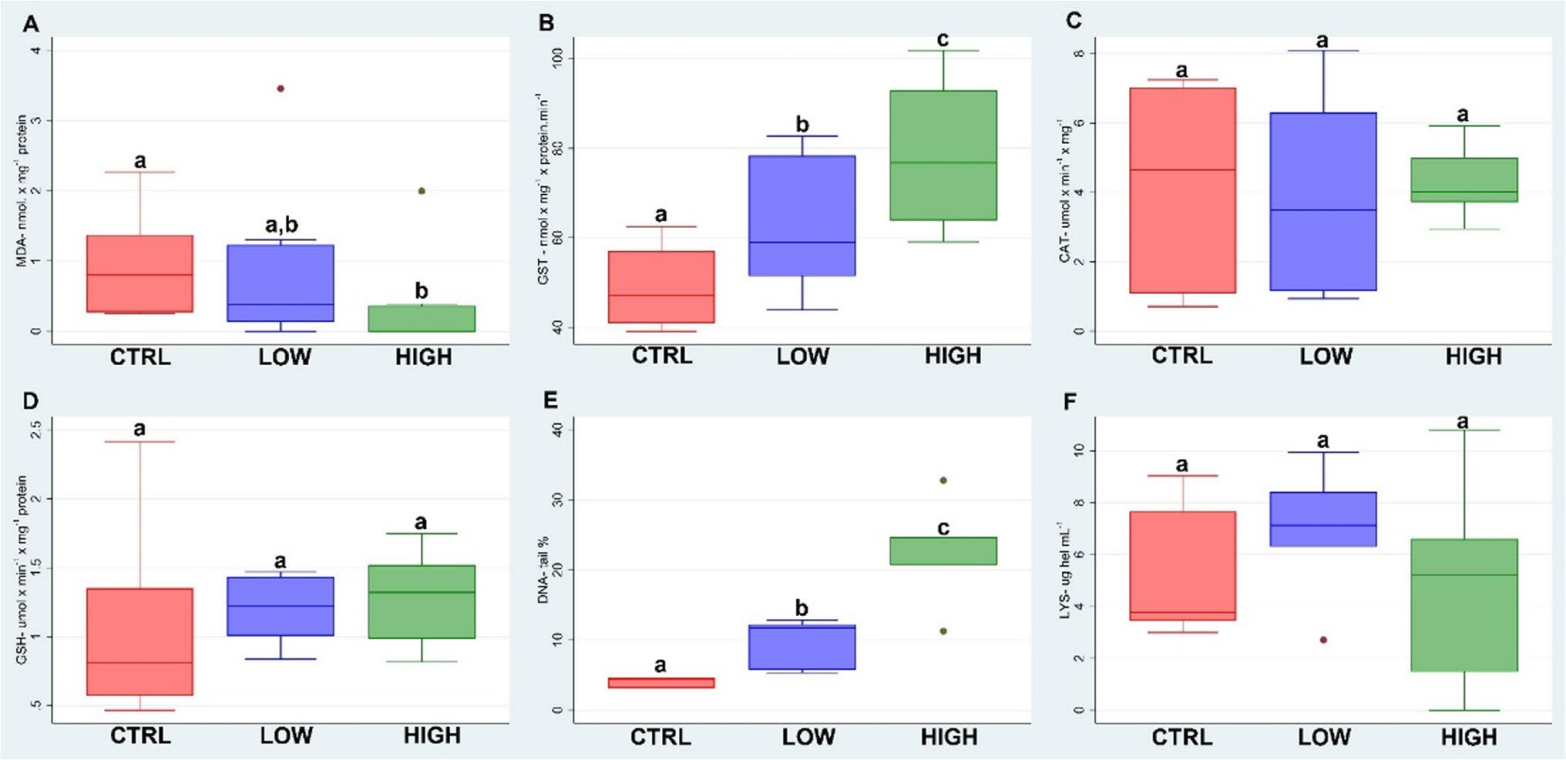
Boxplots (the median, the inter-quartile range and the range of scores) of the biomarkers (lipid peroxidation levels (LPO) (**A**); glutathione S-transferase activity (GST) (**B**); atalase activity (CAT) (**C**); total glutathione levels (GSHt) (**D**); comet assay (**E**); lysozyme activity (LYS) (**F**)) measured in *E. fetida* exposed to the fungicide Prosaro®. Different letters indicate a statistical difference between groups (*p* < 0.05)

Figure 4 reports the biomarker results of the fungicide Prosaro® tested by the filter paper test. The lipid peroxidation (A) showed a decrease in the treatment of 1.25 μg/cm^2^ statistically significant with respect to the control (*p* < 0.05). GST activity (B) was induced in a dose–response manner; statistically significant differences were found in both treatments with respect to control and between the treatments. The same trend was obtained in the GSHt levels (D). The CAT enzyme was not induced in either of the treatments (C). The comet assay (E) showed a significant increase in DNA fragmentation in both treatments with respect to the control (*p* < 0.05). The lysozyme (F) showed higher levels of activity in the 0.75 μg/cm^2^ treatment without statistical difference.

The GST induction can be due to the involvement of this enzyme in the metabolization process of the two main active ingredients of Prosaro®, tebuconazole and prothioconazole. Nevertheless, several studies demonstrated that oxidative stress is one of the main mechanisms of action of triazole fungicides including tebuconazole (Yang et al. 2018; Othmène et al. 2020). Studies with cultured human cells showed that tebuconazole increased the production of ROS (Heusinkveld et al. 2013). Another study on the common carp (*Cyprinus carpio*) exposed to tebuconazole showed increased lipid peroxidation (TBARS) (Toni et al. 2011). These findings are different from our results which could be explained by a strong detoxification effort, deduced from the GST data, that prevents lipid peroxidation. The increased DNA strand break frequency was measured in both treatments as reported by Aktaş et al. (2018), which showed that tebuconazole-based fungicide (Luna experience 400 SC) caused DNA strand breaks and micronucleus (MN) frequency increase in rat liver and blood tissues.

Differently from Icarus®, which did not affect *E. fetida*, Prosaro® fungicide, with two triazole-based active ingredients, amplifies the toxicological effect on *E. fetida* genotoxicity already at the field dose.

## Conclusions

In conclusion, the exposure to field doses of the fungicides Mirador® and Amistar®Xtra was found to cause an imbalance of ROS species in *E. fetida* specimens, leading to the inhibition of the immune system in the case of Amistar®Xtra. Moreover, the presence of the two active ingredients in the Amistar®Xtra and Prosaro® induced significant DNA alterations through two different ways of action. The azoxystrobin-based fungicide (Amistar®Xtra) caused an imbalance of ROS leading to genotoxic effects. The triazoles-base fungicide Prosaro® acted directly on DNA.

The results of this study broaden our knowledge about the effects of pesticide product formulations on earthworms. Moreover, the results showed the inadequacy of current pesticide testing requirements, based on the evaluation of the single individual ingredient and limited only to the active ingredients. This procedure ignores the toxicological risk deriving from the changing of physicochemical and toxicological properties that occur when active ingredients are used in combination and mixed with co-formulant. It would be desirable for European and extra European legislations to ensure that commercial formulations are tested before they are commercialised, to prevent unwanted effects on non-target soil organisms.

## Data Availability

Not applicable.
